# Formant-Frequency Variation and Informational Masking of Speech by Extraneous Formants: Evidence Against Dynamic and Speech-Specific Acoustical Constraints

**DOI:** 10.1037/a0036629

**Published:** 2014-05-19

**Authors:** Brian Roberts, Robert J. Summers, Peter J. Bailey

**Affiliations:** 1 Psychology, School of Life and Health Sciences, Aston University, Birmingham, United Kingdom; 2 Department of Psychology, University of York, Heslington, York, United Kingdom

**Keywords:** speech perception, formant frequency, informational masking, auditory grouping, cocktail party effect

## Abstract

How speech is separated perceptually from other speech remains poorly understood. Recent research indicates that the ability of an extraneous formant to impair intelligibility depends on the variation of its frequency contour. This study explored the effects of manipulating the depth and pattern of that variation. Three formants (F1+F2+F3) constituting synthetic analogues of natural sentences were distributed across the 2 ears, together with a competitor for F2 (F2C) that listeners must reject to optimize recognition (left = F1+F2C; right = F2+F3). The frequency contours of F1 − F3 were each scaled to 50% of their natural depth, with little effect on intelligibility. Competitors were created either by inverting the frequency contour of F2 about its geometric mean (a plausibly speech-like pattern) or using a regular and arbitrary frequency contour (triangle wave, not plausibly speech-like) matched to the average rate and depth of variation for the inverted F2C. Adding a competitor typically reduced intelligibility; this reduction depended on the depth of F2C variation, being greatest for 100%-depth, intermediate for 50%-depth, and least for 0%-depth (constant) F2Cs. This suggests that competitor impact depends on overall depth of frequency variation, not depth relative to that for the target formants. The absence of tuning (i.e., no minimum in intelligibility for the 50% case) suggests that the ability to reject an extraneous formant does not depend on similarity in the depth of formant-frequency variation. Furthermore, triangle-wave competitors were as effective as their more speech-like counterparts, suggesting that the selection of formants from the ensemble also does not depend on speech-specific constraints.

The ability of human listeners to attend selectively to one talker in the presence of others, known as the cocktail party effect, has long interested researchers (e.g., Cherry, 1953). In a recent study, volunteers being prepared for epilepsy surgery were presented with a mixture of speech from two talkers, one male and one female, and multielectrode arrays were used to record their cortical responses while they attended one or other voice (Mesgarani & Chang, 2012). Speech spectrograms reconstructed from these responses revealed the salient spectral and temporal features of the attended talker, as if the representation of the interfering speech was partly suppressed and listeners were hearing something akin to a “cleaned up” version of the target speech. In this example, well established auditory grouping cues are likely to have been important, such as differences in fundamental (F0) frequency (e.g., Bird & Darwin, 1998; Brokx & Nooteboom, 1982), but a full account of the perceptual bases on which the effective target-to-interferer ratio for speech mixtures can be improved remains elusive. In particular, few studies have explored the role of the time-varying properties of speech in separating a target voice from an interfering voice.

Spectral prominences in speech, called formants^1^, are perceptually important because they convey articulatory information about vocal tract shape and its change over time. Hence, knowledge of formant frequencies and their change over time is of considerable benefit to listeners trying to understand a spoken message (e.g., Roberts et al., 2011). When more than one talker is speaking at once, choosing and grouping together the right set of formants from the mixture is critical for intelligibility, yet few studies have focused specifically on across-formant integration and segregation, or on the attentional selection of a subset of formants from a stimulus ensemble. The relative contributions of general-purpose grouping principles (“primitives;” Bregman, 1990), and of speech-specific factors (Remez, Rubin, Berns, Pardo, & Lang, 1994) to the perceptual coherence of speech remain unclear, and the critical acoustical correlates of the latter—if they exist at all—remain almost unknown (see, e.g., Darwin, 2008). The parametric manipulations possible with simplified speech signals make them attractive stimuli with which to explore these issues. In the experiments reported here, we have used synthetic-formant analogues of natural sentence-length speech.

We used the second-formant competitor (F2C) paradigm, in which listeners must reject a single extraneous formant to optimize recognition of short sentences (Remez et al., 1994; Roberts, Summers, & Bailey, 2010; Summers, Bailey, & Roberts, 2010). The core of this paradigm is the dichotic presentation (i.e., to opposite ears) of the first and second formants (F1 and F2), together with the second-formant competitor (F2C)—an alternative possibility for the second formant—in the same ear as F1; the third formant (F3) is also presented only in one ear. This paradigm has four particular advantages as an experimental tool. First, the factors governing perceptual organization are generally revealed most clearly where competition operates (e.g., Barker & Cooke, 1999; Darwin, 1981). Second, this type of dichotic configuration (e.g., left ear = F1+F2C; right ear = F2+F3) minimizes energetic masking of the target formants—that is, masking caused by the energy of the competitor formant swamping that of the target formants (especially F2) in the auditory-nerve response to the stimulus. Hence, the effect of the competitor arises primarily through informational masking (see, e.g., Durlach et al., 2003a), which is central in origin and can be defined operationally as interference beyond that attributable to energetic masking. Third, the lateralization cues for this configuration favor the fusion of F1 with F2C rather than with the target F2 (cf. Culling & Summerfield, 1995), and hence might be expected to increase the impact of the competitor formant (its efficacy). Fourth, sentence intelligibility provides a straightforward measure of competitor efficacy.

Although it must be acknowledged that the use of simplified speech analogues and the dichotic presentation of formants inevitably compromise some aspects of ecological validity, it is important to note that speech is a sparse signal on a frequency–time representation like a spectrogram. This is because speech consists mainly of discrete harmonics, the amplitudes of which vary gradually with frequency and are maximal near the formant center frequencies. In addition, there are silent or low-amplitude intervals during the vocal-tract closures associated with the production of stop consonants and affricates. Therefore, when two different speech signals of similar overall level are mixed together, each local frequency–time region is usually dominated by one or other of the signals (e.g., Darwin, 2008). Hence, when there are two talkers, energetic masking usually affects only parts of the target speech, limited in both frequency and time (e.g., Cooke, 2006). Except for highly adverse signal-to-noise (S/N) ratios, this means that separating two voices is primarily a problem of assigning readily detectable frequency–time regions to the correct source rather than one of detecting parts of the target signal (e.g., Darwin, 2008), and so the impact of an interfering voice on the intelligibility of target speech often arises mainly through informational masking (e.g., Brungart et al., 2006).

The F2C paradigm was created by Remez et al. (1994) with the specific intention of investigating across-formant grouping. There are two ways in which the competitor might act to reduce sentence intelligibility by affecting the perceptual organization of the formant ensemble. First, a failure of segregation (partial or complete) may allow F2C to contribute to the perceptual estimation of F2 and thus to dilute or corrupt the phonetic information provided by the target F2, leading to errors in word recognition (Roberts et al., 2010). This account is related to an earlier finding that identification of a synthetic consonant–vowel (CV) syllable presented in one ear is influenced systematically by the direction of isolated F2 transitions presented in the opposite ear (Porter & Whittaker, 1980). Second, F1 may undergo spatial capture by F2C, thus impairing the across-ear integration of the phonetic information carried by F1 with that carried by F2. To date, the results of our studies using this paradigm have been interpreted mainly in terms of changes in across-formant grouping. In the current study, we consider the impact of competitors on sentence intelligibility in a broader context.

In general terms, it is often useful to characterize informational masking as a failure of either auditory object formation or object selection (see, e.g., Shinn-Cunningham, 2008). Failures in object selection can occur because attention is directed to the wrong object—because the listener either does not know which features to attend or the features of the target and interferer are not distinct enough (e.g., Darwin et al., 2003)—or because the salience of the interferer makes it difficult to inhibit the distracting information (e.g., Conway et al., 2001). As such, informational masking may arise not only from the effects of grouping and segregation but also from limitations on a range of other perceptual and cognitive processes, such as attention, memory, and general processing capacity (see Kidd et al., 2008, for a review). Indeed, one might expect most or all of these limitations to contribute in the context of a complex and dynamic broadband signal like speech (see Mattys et al., 2012, for a review). Hence, identifying the underlying causes of changes across conditions in the impact of an extraneous formant on intelligibility is not necessarily straightforward.

Remez et al. (1994) first introduced the F2C paradigm in a study aimed at exploring the perceptual organization of sine-wave analogues of speech (Bailey et al., 1977; Remez et al., 1981). They showed that an F2C created by time-reversing a tonal F2 was an effective competitor, but a pure tone of constant frequency and amplitude was not, suggesting that acoustic variation was critical for competition. Roberts et al. (2010) explored aspects of this variation using separate manipulations of the frequency and amplitude contours of competitor formants to tease apart their impact on the intelligibility of sine-wave speech. All F2Cs with time-varying frequency contours (time reversed or spectrally inverted) were highly effective competitors, regardless of their amplitude characteristics, but F2Cs with constant frequency contours were ineffective. These results led to the proposal that it is the variation of the formant-frequency contours—their modulation patterns—that are critical for across-formant grouping (Roberts et al., 2010); a similar argument can be made in terms of attentional selection of the appropriate set of formants from the stimulus ensemble. Regardless of whether the interference caused by F2C arises from a failure of object formation, incorrect object selection, or from limits on processing capacity, the relationship between formant-frequency variation in an extraneous formant and its impact on intelligibility might arise in principle either from speech-specific properties, as proposed by Remez et al. (1994), or from more general aspects of the auditory processing of time-varying broadband stimuli.

What aspects of formant-frequency variation might be important factors influencing the impact of an extraneous formant on speech intelligibility? Summers, Bailey, and Roberts (2012) explored the role of the rate of formant-frequency variation, using synthetic-formant speech and a variant of the formant-competitor paradigm involving a pair of time-reversed frequency contours for the competitor formants (F2C+F3C). Although the acoustical concomitants of changes in natural speech rate are many and varied, they commonly include changes in the rate of formant-frequency variation (e.g., Weismer & Berry, 2003). Therefore, in principle, differences in the rate of formant-frequency variation between talkers might provide a basis for the appropriate grouping and segregation of formants. A hypothesis based on grouping by similarity in this dynamic property^2^ would predict that the impact of a competitor on intelligibility is rate tuned, such that maximum interference occurs when the rate of formant-frequency variation for the competitor is most like that for the target formants. This prediction holds irrespective of whether the competitor (F2C+F3C) acts by contributing to the perceptual estimation of F2 and F3 or by reducing the likelihood of across-ear integration of F1 with F2 and F3. Alternatively, it may be that faster variations are more disruptive, such that interference is proportional to the rate of formant-frequency variation in the competitor. If both factors operate, and the segregation of F2C is incomplete, one might expect to observe a sloping function with a local minimum for the case where all formants in the ensemble share a common rate of frequency variation.

Summers et al. (2012) evaluated these hypotheses by manipulating the rate of formant-frequency change in the competitor relative to that for the target formants (baseline). They found that the impact of F2C+F3C on intelligibility rose gradually and progressively as the rate of frequency variation in the competitor formants was increased, for rates up to at least twice baseline. Given that there was no minimum in intelligibility when competitor rate matched that of the target formants, Summers et al. (2012) concluded that competitor efficacy is not tuned to the rate of variation in the target sentences, but instead depends on the overall rate of formant-frequency change in the competitor (i.e., higher rates increase competitor efficacy). This pattern is consistent with the hypothesis that faster variation in the frequency contours of extraneous formants is more disruptive. The results suggest that differences in speech rate as such do not provide significant cues for the across-frequency grouping of formants when segregating the speech of concurrent talkers. This outcome contrasts with the effects of a well-characterized primitive grouping cue, ΔF0, for which competitor efficacy is greatest when the competitor and target formants share a common F0 (Summers et al., 2010; see also Darwin, 1981; Gardner et al., 1989). Consistent with the results for sine-wave speech (Roberts et al., 2010), rate of amplitude change in the competitor (rate-adjusted natural envelope vs. constant amplitude) had no discernible effect on intelligibility.

The experiments reported here extend our exploration of the effects of formant-frequency variation on the interference caused by an extraneous formant, and the extent to which this interference might arise from failures of auditory object formation, incorrect object selection, or limits on processing capacity. We examined the effect on intelligibility of manipulating the *depth* and the *pattern* of variation in the formant-frequency contour of F2C, relative to that for the target formants. The range of frequencies that a formant traverses during natural speech is determined primarily by the extent of the movements of the articulators governing the size of the vocal tract cavity associated with that formant. As a simple illustration of this principle, the second formant—typically associated with the front cavity—falls in frequency as the tongue moves back in the vocal tract from a forward position, because this maneuver (together with others, such as lip rounding) leads to a change in front cavity size from small to large, as for the movement between articulator positions for the vowels /i/ and /u/ (see, e.g., Stevens, 1998). In an articulatory maneuver of this kind, the magnitude of the frequency change—the depth of formant-frequency variation—is influenced by how extreme are the initial and final articulator positions (Lindblom & Sundberg, 1971).

The depth of formant-frequency variation in our stimuli was manipulated using a linear scale factor. To investigate the effect of the pattern of variation, some conditions used F2Cs whose frequency contours were created by spectral inversion of F2, and hence retained a pattern of acoustic variation that was relatively speech-like, whereas others used F2Cs with regular and arbitrary frequency contours that were not plausibly speech-like. Taken together, the results of these experiments suggest that across-formant grouping and selection do not depend on either similarity in the dynamic properties of formant-frequency contours or the articulatory plausibility of this frequency variation. Rather, the interference attributable to the extraneous formant increases as its depth of formant-frequency variation increases. These outcomes are interpreted in the contexts of auditory grouping and informational masking, and their role in speech perception.

## General Method

### Stimulus Synthesis

The stimuli for the main parts of the three experiments in the current study were synthetic-formant analogues of speech derived from recordings of 92 sentences spoken by a British male talker of “Received Pronunciation” English. The text for these sentences was provided by M. Patel and R. P. Morse (personal communication, 2010) and consisted of variants created by rearranging words from the Bamford-Kowal-Bench (BKB) sentence lists (Bench et al., 1979). To enhance the intelligibility of the synthetic analogues, the sentences used were semantically simple and selected to contain ≤ 25% phonemes involving vocal tract closures or unvoiced frication. A set of keywords was predesignated for each sentence—there is no generally agreed definition of what constitutes a keyword and so the choice is somewhat arbitrary, but most keywords were content words. The stimuli for the training sessions were derived from a corpus of sentences taken from commercially available recordings of the Harvard sentence lists (IEEE, 1969) and were spoken by a different talker. These sentences were also selected to contain ≤ 25% phonemes involving closures or unvoiced frication.

For each sentence, the pitch contour and the frequency contours of the first three formants were estimated from the waveform automatically every 1 ms from a 25-ms-long Gaussian window, using Praat software (Boersma & Weenink, 2010). In practice, the third-formant contour often corresponded to the fricative formant rather than F3 during phonetic segments with frication; these cases were not treated as errors. Gross errors in automatic estimates of the three formant frequencies were hand-corrected using a graphics tablet; artifacts are not uncommon and manual postprocessing of the extracted formant tracks is often required (Remez et al., 2011). Amplitude contours corresponding to the corrected formant frequencies were extracted automatically from the spectrograms for each sentence. Synthetic-formant analogues of each sentence were created using these frequency and amplitude contours (or rescaled versions of the frequency contours, see below) to control three parallel second-order resonators whose outputs were summed. The excitation source for the resonators was a periodic train of simple excitation pulses modeled on the glottal waveform, which Rosenberg (1971) has shown to be capable of producing synthetic speech of good quality. The 3-dB bandwidths of the resonators corresponding to F1, F2, and F3 were set to constant values of 50, 70, and 90 Hz, respectively. In the main parts of the experiments, the excitation source was monotonous (F0 = 140 Hz), but the stimuli for the training sessions were generated using the pitch contours extracted from the original recordings.

Rescaling of the formant-frequency contours was performed on a log-frequency scale. In effect, each contour was converted to a vector specifying, frame by frame, the frequency as a deviation from the geometric mean frequency of the whole track. The depth of frequency variation around the geometric mean was then adjusted by multiplying the vector using a scale factor in the range 0 (i.e., constant at the geometric mean frequency) to 1 (i.e., original depth). Intermediate values act to reduce formant excursions away from the mean; hence, the manipulation may be referred to as the “formant squash.” The acoustical effect of this squash is similar to that of physically constraining the extent and rate of excursions made by the main articulators—the tongue, lips, and jaw—away from their average positions; given that utterance duration is unaltered, this has the effect of reducing the extent and velocity of formant-frequency variation. In formal terms, the rescaled frequency for each formant at time *t*, *s*(*t*), is given by:

$$\log\;s(t)=\log\;g+x\left(\log\;\frac{f(t)}{g}\right)$$ 1

where *x* (0 ≤ *x* ≤ 1) is a proportional scale factor determining the maximum possible frequency range (depth of variation), *f*(*t*) is the formant frequency at time *t*, and *g* is the geometric mean of the whole formant-frequency contour. In the current study, the frequency contours of the three target formants were adjusted in parallel; hence, they were always scaled by the same factor within any given condition.

In Experiment 1, the target formants were presented diotically (i.e., all formants to both ears). In Experiments 2 and 3, they were instead presented dichotically and were usually accompanied by an extraneous formant. This extraneous formant was intended to act as a competitor for F2; its frequency contour was derived from that for the target F2. These stimulus configurations, and synthesis of the competitors, are described in detail in the context of the appropriate experiments. All speech analogues were synthesized using MITSYN (Henke, 2005; see www.mitsyn.com) at a sample rate of 22.05 kHz and with 10-ms raised-cosine onset and offset ramps. They were played at 16-bit resolution over Sennheiser HD 480-13II earphones (Hannover, Germany) via a Turtle Beach Santa Cruz sound card (Valhalla, NY), programmable attenuators (Tucker-Davis Technologies, TDT PA5; Alachua, FL), and a headphone buffer (TDT HB7). Output levels were calibrated using a sound-level meter (Brüel & Kjaer, Type 2209; Nærum, Denmark) coupled to the earphones by an artificial ear (Type 4153).

### Listeners

Volunteers were first tested using a screening audiometer (Interacoustics AS208; Assens, Denmark) to ensure that their audiometric thresholds at 0.5, 1, 2, and 4 kHz did not exceed 20 dB HL. All volunteers who passed the audiometric screening took part in a training session designed to improve the intelligibility of the synthetic-formant speech analogues (see below). There were 88 volunteers in total, of whom 60 passed the training and took part in the main experiments. There were also occasional exclusions based on posttraining performance, as described below. To our knowledge, none of the listeners had heard any of the sentences used in the main parts of the experiments in any previous study or assessment of their speech perception. All listeners were native speakers of English, usually British English, and gave informed consent. The research was approved by the Aston University Ethics Committee.

### Procedure

During testing, listeners were seated in front of a computer screen and a keyboard in a sound-attenuating chamber (Industrial Acoustics 1201A; Winchester, United Kingdom). Each experiment consisted of a training session followed by the main session; Experiment 2 also had a short follow-up test of intelligibility immediately afterward (see below). These experiments typically took from about 45 min (Experiments 1 and 3) to about an hour (Experiment 2) to complete all parts; listeners were free to take a break whenever they wished. In each part of every experiment, stimuli were presented in a new quasi-random order for each listener.

Depending on the experiment, there were either 40 or 45 trials in the training session, which was intended to familiarize listeners with the synthetic stimuli and to enhance their intelligibility. On each of the first 10 trials, participants heard the synthetic version (S) and the original recording (clear, C) of a given sentence in the order SCSCS; no response was required but participants were asked to listen to these sequences carefully. On each of the remaining trials, listeners first heard the synthetic version of a given sentence, which they were asked to transcribe using the keyboard. They were allowed to listen to the stimulus up to a maximum of six times before typing in their transcription. After each transcription was entered, feedback to the listener was provided by playing the original recording (44.1 kHz sample rate) followed by a repeat of the synthetic version. Davis, Johnsrude, Hervais-Adelman, Taylor, and McGettigan (2005) found this strategy to be an efficient way of enhancing the perceptual learning of speech-like stimuli.

A mean criterion of ≥ 50% keywords correct across the last 20 training trials was set for a listener to continue on to the main session. In the main session, as for training, participants were able to listen to each stimulus up to six times without time limit before typing in their transcription. However, in the main session they did not receive feedback of any kind on their responses.

### Data Analysis

For each listener, the intelligibility of each stimulus was quantified in terms of the percentage of keywords identified correctly; homonyms were accepted. The stimuli for each condition comprised from four (Experiment 1) to six sentences (Experiments 2 and 3). Given the variable number of keywords per sentence (2–5), the mean score for each listener in each condition was computed as the percentage of keywords reported correctly giving equal weight to all the keywords used. Following the procedure of Roberts et al. (2010), we classified responses using tight scoring, in which a response is scored as correct only if it matches the keyword exactly (see Foster et al., 1993).

Typed responses were also converted automatically into phonemic representations using eSpeak (Duddington, 2008). This software uses a pronunciation dictionary and a set of generic pronunciation rules for English orthography to generate phonemic representations of the input text. The dictionary contains representations for ∼2,000 of the most common English words; a complex set of inbuilt rules is used to generate representations of words not in the dictionary, or of nonwords and misspelled words. Obvious spelling or typographical errors (e.g., a reversal of “e” and “i” in “received”) were corrected manually before the transcription was passed to eSpeak for phonemic analysis. Nonetheless, it should be acknowledged that there will have been occasional errors in the automatic conversion where the context could lead to differing pronunciation (e.g., rendering the text string “read” as either “red” or “reed”).

The mean percentage of phonemic segments correctly identified across *all words* in the sentences was computed using HResults, part of the HTK software (Young et al., 2006). HResults finds an optimal alignment between the phonemic segments of the original sentence and its transcription by inserting and removing segments in the transcription. The mean percentage of phonemic segments correctly identified—here referred to as the phonemic score—is defined as 100 × (number of correctly aligned phonemes)/(number of phonemes in the original sentence). In effect, tight scoring and phonemic scoring represent the lower and upper limits of the intelligibility measures that can be computed for the test sentences used. In addition to computing phoneme scores, it is also possible to examine changes across conditions in the likelihood of making particular classes of phonemic response. In principle, this may be useful for elucidating more precisely the ways in which particular manipulations affect intelligibility.

## Experiment 1

If the auditory system is able to group or select acoustic elements on the basis of similarity in their dynamic properties, one might predict that the impact of a competitor on intelligibility will be tuned such that maximum interference will occur when the depth of formant-frequency variation for the competitor is most like that for the target formants. Alternatively, it may be that larger variations are more disruptive, such that interference is proportional to the average depth of formant-frequency variation in the competitor. To distinguish between these hypotheses, it is necessary to include conditions in which the depth of formant-frequency variation for the competitor is greater, as well as smaller, than that for the target formants. However, if the average depth of formant-frequency variation is maintained in the target sentences, this can only be achieved by including conditions in which F2C frequency variation is expanded beyond the range of variation in the natural utterances. The main problem is the limited scope for expansion available before the experimental stimuli violate the close-approach constraint between F2C and F1 (as described below, in the Method for Experiment 2). An additional difficulty is that any evidence of reduced competitor efficacy as F2C frequency variation is expanded beyond the natural range might reflect a reduction in the plausibility of the articulatory gestures implied by the competitor (i.e., of its speech-like variation) rather than grouping or selection by similarity.

Experiment 1 examined the effect of scaling down the depth of formant-frequency variation in the target sentences to establish whether substantial compression was possible without unduly compromising their intelligibility. If so, it becomes possible to examine the effect of scaling F2Cs to have variation in their formant-frequency contours greater than that of the target formants without the difficulties associated with expansion beyond the natural range. To our knowledge, only one previous study has measured the effects of squashing the extent of formant-frequency variation on the intelligibility of sentence-length stimuli. Remez and Rubin (1992) used a constant-F0 source to generate sets of synthetic-formant analogues of a sentence differing only in average depth of formant-frequency variation (i.e., the amplitude contour was not adjusted). Scale changes were made relative to the nominal formant-frequencies of a standard tube model of the adult male vocal tract (i.e., F1 = 500 Hz, F2 = 1,500 Hz, and F3 = 2,500 Hz), rather than to the geometric mean frequency of each formant. Stimuli were scaled in 10% steps (from 10% to 100%) and presented in single-sentence blocks, in order of increasing frequency variation (and hence ease of intelligibility).

The resulting psychometric function suggested that intelligibility declines quite steeply once the scaling factor falls below 80%. However, this outcome may not generalize to circumstances where a large set of sentences is used (Remez and Rubin used only two) and where all sentences and scaling factors are randomized together within the same block of trials. Hence, better characterization of the relationship between formant-frequency variation and intelligibility in the absence of extraneous sounds is also of interest in its own right.

### Method

#### Stimuli and conditions

**Figure 1 fig1:**
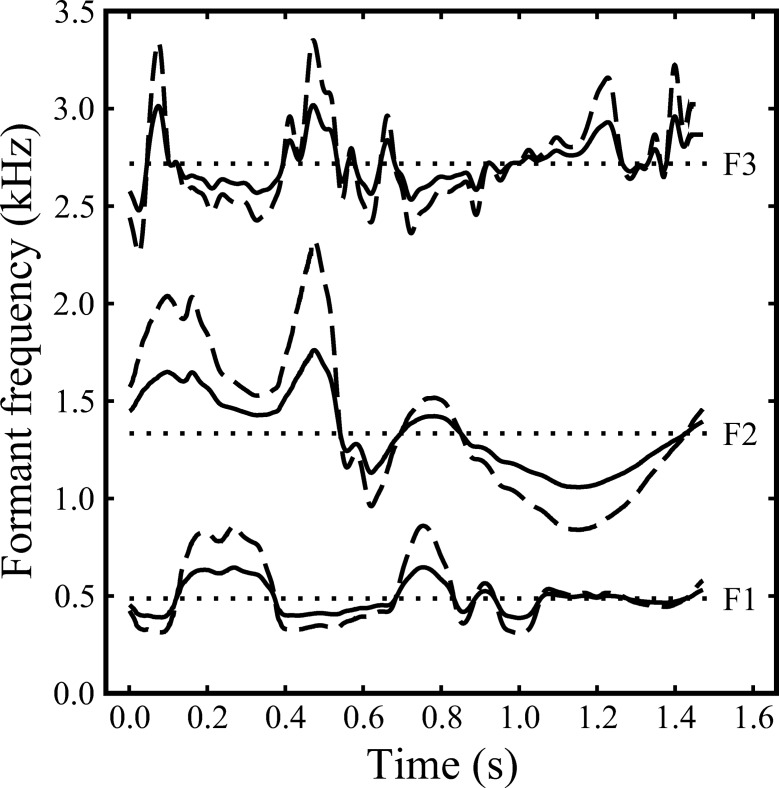
Stimuli for Experiment 1: Schematic illustrating the use of scale factors to control the depth of formant-frequency variation in three-formant synthetic speech. Using the example sentence “The cat ran along,” the frequency contours of F1, F2, and F3 are shown for the cases where the scale factor was 100% (i.e., not adjusted; dashed line), 50% (solid line), and 0% (dotted line). Depth of formant-frequency variation was controlled by applying a common scale factor to a set of values for each formant, representing its frequency contour in terms of deviations from the geometric mean frequency on a log scale (Equation 1). Hence, for the 0% case, the frequency of each formant was set to be constant at the geometric mean frequency for that formant track. The full set of scale factors used ranged from 100% to 0%, in steps of 10%. Note that only the formant-frequency contours were adjusted in this way; formant amplitude contours (not shown here) were always presented without adjustment.

The stimuli comprised synthetic three-formant analogues of 44 sentences, presented diotically and without competitors. There were 11 conditions in the main experiment, which differed only in the magnitude of the common scale factor applied to the frequency contours of all three formants. Eleven versions of each sentence were created by changing the scale factor from 100% to 0% (constant at the geometric mean) in 10% steps; the amplitude contours of the formants were not changed by this manipulation. A schematic showing the effect of scaling the formant-frequency contours of the stimuli is shown in Figure 1. For each listener, the 44 sentences used were divided equally across the 11 conditions (i.e., four per condition), such that there were always 12 or 13 keywords per condition. Allocation of sentences was counterbalanced by rotation across each set of 11 listeners tested; hence, the experiment required a multiple of 11 listeners to produce a balanced dataset. There were 40 sentences used in the training session, for which all the formants were scaled to 100% depth.[Fig-anchor fig1]

#### Listeners and procedure

Twenty-two listeners (eight males) passed the training and completed the experiment; there were no further exclusion criteria. Eleven of these listeners also took part in Experiment 3. Listeners were drawn from an undergraduate and postgraduate university population, and had a mean age of 22.4 years (range = 18.5–33.3; *SD* = 4.8 years). In both the main and training sessions, all stimuli were presented diotically at a reference level (long-term average) of 72 dB SPL.

### Results

**Figure 2 fig2:**
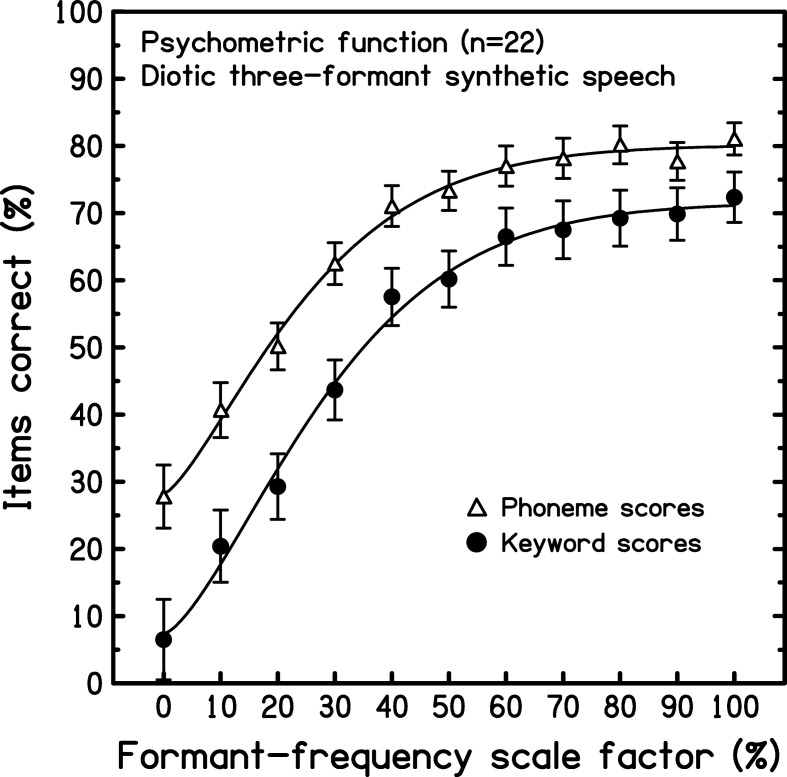
Results for Experiment 1: Psychometric function showing the influence of depth of formant-frequency variation on the intelligibility of three-formant analogues of the target sentences, under diotic presentation. The depth of variation in each formant-frequency contour was adjusted, using a common scale factor, to one of a range of values about its geometric mean (100% to 0% [i.e., constant], in steps of 10%). Mean scores and intersubject standard errors (*n* = 22) are shown for keywords (filled circles) and phonemes (open triangles). Each set of scores has been fitted using a Weibull function (solid lines), for which the equation is$\Psi (x)=\gamma +(1-\gamma -\lambda )\left(1-\exp\left(-{\left(x/\alpha \right)}^{\beta }\right)\right)$. The parameter values for the fit to the keyword scores are: γ = 0.073 (guess rate), λ = 0.284 (lapse error rate), α = 32.978 (point of inflection), and β = 1.455 (slope). The parameter values for the fit to the phonemic scores are: γ = 0.278, λ = 0.201, α = 28.120, and β = 1.364. These fits are good: *r*^2^(9) = 0.998 (keywords) and 0.995 (phonemes). Note that using a scale factor of 50% has only a modest impact on intelligibility.

Figure 2 shows the mean percentage scores (and intersubject standard errors) across conditions in terms of keywords (filled circles) and phonemes (open triangles) correctly identified. Each set of scores has been fitted, using a Weibull function (Wichmann & Hill, 2001), to give a psychometric function describing the influence of depth of formant-frequency variation on the intelligibility of three-formant analogues of the target sentences. As would be expected, both functions are similar in form but the mean phoneme scores are consistently higher than their keyword counterparts. Although keyword scores were slightly more variable than phoneme scores across listeners, this effect was offset by the greater range of scores across scale factors for mean keyword than for mean phoneme scores (65.8% vs. 53.3%). Moreover, the nature of phonemic scoring is such that chance factors become increasingly important as intelligibility declines; intelligibility is typically limited when speech analogues are presented dichotically and in the presence of competitors, as in Experiments 2 and 3. Hence, as in our previous studies (Roberts et al., 2010; Summers et al., 2010, 2012), keyword scores were taken as the primary measure of intelligibility; all statistical analyses reported used these scores.[Fig-anchor fig2]

A one-way within-subjects analysis of variance (ANOVA) on the keyword scores showed a highly significant effect of condition on intelligibility, *F*(10, 210) = 40.651, *p* < .001, η^2^ = 0.659^3^. Only a limited number of pairwise comparisons (two-tailed) were required from the large set of possible comparisons, and so they were computed using the restricted least significant difference test (Snedecor & Cochran, 1967; Keppel, 1991). The lower half of the psychometric function was relatively steep; all pairwise comparisons between adjacent means were significant over the set of scale factors from 0% to 30% (range: *p* = .018 – < .001). Conversely, the upper half of the psychometric function was shallow; compared with the full-scale case, the formant-frequency contours had to be scaled down to 50% depth before the fall in keyword recognition became significant, mean scores = 72.3% versus 60.2%; *t*(21) = 2.36, *p* = .028. The corresponding change in mean phoneme scores over this range was also modest (81.1% vs. 73.3%).

### Discussion

The pattern observed differs from that reported by Remez and Rubin (1992). They found that scaling down the extent of formant-frequency variation in their two test sentences to 50% depth caused syllable recognition (their measure of intelligibility) to fall from above 80% to below 20% correct. Most probably, the discrepancy can be attributed to limitations of their method, particularly the presentation of a single sentence in order of increasing frequency variation, and hence increasing ease of intelligibility, rather than in random order. The finding that depth of formant-frequency variation in the target sentences can be squashed by half from its natural extent with relatively little loss of intelligibility allowed the use of stimuli scaled in this way in our subsequent experiments.

As already noted, the depth of formant-frequency variation in natural speech is the principal correlate of the extent to which the articulators move from their average positions during speech production. However, it should be acknowledged that the changes in articulation typically associated with changes in the depth of formant-frequency variation often have other acoustical correlates, such as changes in the rate of formant-frequency variation (e.g., Ferguson & Kewley-Port, 2007; Fourakis, 1991) and the shape of formant-frequency trajectories (e.g., Erickson, 2002; Lindblom et al., 2007). Nonetheless, the finding that the intelligibility of our stimuli remains fairly robust across the upper half of the range of adjustment suggests that our intentionally simplistic approach of using a linear scale factor to control the depth of formant-frequency variation is a reasonable one.

## Experiment 2

Remez (1996, 2001) reported that reducing the frequency variation in a competitor created by time-reversing a tonal F2 lessened its impact on the intelligibility of sine-wave speech. We extended this approach to synthetic-formant speech and refined it by manipulating the depth of variation in the frequency contour of a time-varying F2C, relative to that for the target formants, in a context where every competitor had a constant-amplitude contour matched to the root mean square (RMS) power of the corresponding F2. Experiment 2 was intended to distinguish between two hypotheses. If differences in the dynamic properties of formants cause them to segregate from one another, or can provide a basis for attentional mechanisms to select a subset of formants from an ensemble, the impact of a competitor on intelligibility will be tuned such that interference will be greatest when the depth of formant-frequency variation for the competitor is most similar to that for the target formants. If, however, larger frequency variations are more disruptive to the extraction of phonetic information from the target formants, interference will instead be proportional to the average depth of formant-frequency variation in the competitor. If both factors contribute, there should still be evidence of a local maximum in interference when the competitor and target formants are matched for depth.

The results of Experiment 1 indicated that scaling the frequency contours of the target formants to 50% depth had relatively little effect on intelligibility. Hence, we were able to use three-formant analogues of the target sentences whose formant-frequency contours were scaled to 50% depth. In our earlier study of the perceptual effects of the rate of formant-frequency variation, Summers et al. (2012) used competitors comprising a pair of formants (F2C+F3C), with the aim of increasing efficacy in the context of synthetic-formant analogues (cf. Summers et al., 2010). In Experiments 2 and 3 reported here, we used a single competitor formant to avoid the potential complications arising from squashing the frequency variation in more than one formant contour. Nonetheless, we anticipated that the efficacy of single-formant competitors would be adequate owing to the use of sentences for which all three target formants were scaled to 50% depth.

As an additional measure to ensure adequate competitor efficacy, we used F2Cs with inverted rather than time-reversed frequency contours. Spectral inversion is a manipulation originally devised by Blesser (1972) and continues to be used in contexts where unintelligible stimuli with speech-like variation are required (see, e.g., Scott et al., 2000). Inverted F2Cs have not previously been tested in the context of synthetic-formant speech; their use here was motivated by the suggestion from earlier research using sine-wave analogues that an inverted F2C may be more effective than a time-reversed F2C (Roberts et al., 2010). In that study, the frequency contour of F2 was inverted about the spectral centroid for that formant, whereas here inversion was about the geometric mean frequency. This change was to ensure a closer match in the dynamic properties of the frequency contours for F2 and F2C, given the already established need to control for the effects of rate of formant-frequency variation (Summers et al., 2012). Specifically, the geometric mean is a better pivot for frequency variation in a formant track, because the spectral centroid is also influenced by amplitude variation.

### Method

#### Stimuli and conditions

**Figure 3 fig3:**
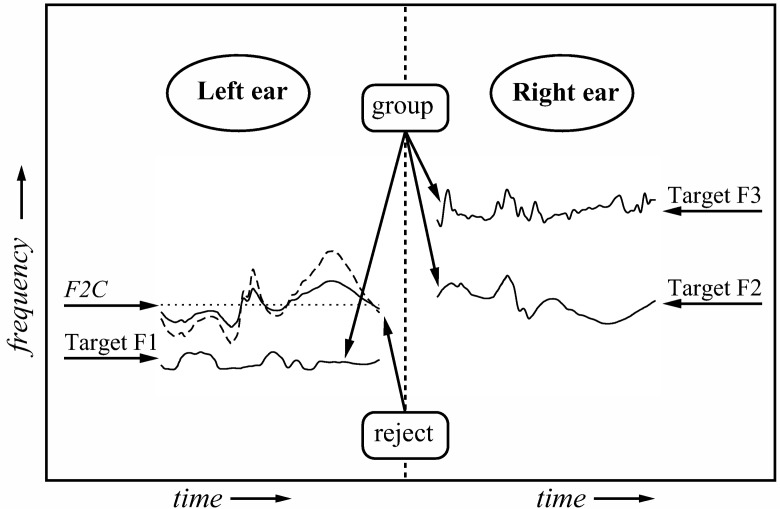
Stimuli for Experiment 2: Schematic illustrating the dichotic configuration used. The left ear receives F1 of the example sentence “The cat ran along;” the right ear receives F2 and F3. A scale factor of 50% was applied to the frequency contour of each target formant, relative to the geometric mean frequency of that formant track. The second-formant competitor (F2C), whose frequency contour was derived from that for F2 by inversion about the geometric mean, is presented in the same ear as F1. The depth of frequency variation in F2C was controlled relative to that for the unscaled target F2. Illustrated here are F2C frequency contours for the cases where the scale factor was 100% (dashed line), 50% (i.e., matching the scale factor for the target formants; solid line), and 0% (dotted line); for the 0% case, the frequency was constant at the geometric mean frequency. The full set of scale factors used ranged from 100% to 0%, in steps of 25%. Note that the amplitude contours (not shown here) of the target formants were always presented without adjustment. The amplitude contour of each F2C was set to a constant value corresponding to the RMS power of the amplitude contour for the target F2.

The stimuli comprised synthetic analogues of 42 sentences; these were a subset of the sentences used in Experiment 1. In the main part of the experiment, the target formants were presented in a dichotic configuration (left ear = F1; right ear = F2+F3; cf. Rand, 1974). Note that this arrangement has an advantage over that used by Remez et al. (1994), in that competitors can be added to the left-ear input without risk of appreciable energetic masking of any of the target formants (Roberts et al., 2010; Summers et al., 2010, 2012). Figure 3 illustrates the stimulus configuration used when the three target formants were accompanied by a competitor. In previous studies, we have demonstrated that there are no appreciable ear-dominance effects for sentence-length utterances in the context of the dichotic F2C paradigm (Roberts et al., 2010; Summers et al., 2010). Therefore, we did not counterbalance for ear of presentation in the dichotic configurations used in Experiments 2 and 3.[Fig-anchor fig3]

A set of F2 competitors was created for each sentence in the main experiment. The excitation source (Rosenberg pulses), F0 frequency (140 Hz), and 3-dB bandwidth (70 Hz) were identical to those used to synthesize the target F2. The frequency contour of each F2C was created by inverting the frequency contour of the target F2 about its geometric mean on a log scale and applying a range of scale factors to the inverted contour (see General Method). Note that spectral inversion about the geometric mean, like time reversal, is an automatic control—both manipulations preserve the rate and depth of frequency variation found in the target F2 contour. Stimuli were selected such that the nominal frequency of F2C was always ≥ 80 Hz from that of F1 at any moment in time. Hence, there were no crossovers of formant tracks or approaches close enough to cause audible interactions between corresponding harmonics exciting different formants.

The amplitude contour of each F2C was set to a constant value corresponding to the RMS power of the amplitude contour for the target F2. Our previous research has shown that the efficacy of a competitor does not depend on the modulation characteristics of its amplitude contour, either for sine-wave speech (Roberts et al., 2010) or for synthetic formant analogues (Summers et al., 2012). One consequence of setting the F2C amplitude contour to a constant nonzero value should be noted. The formant-frequency estimation algorithm that extracts the original F2 contour is prone to error when energy in the original signal is low (e.g., during periods of relative vocal tract occlusion); such errors are not evident in the synthesized sentences because estimates of the formant-amplitude parameter are correspondingly low at those moments, and so these errors were not always corrected. Errors in formant-frequency estimates are more likely to be realized in F2Cs that are synthesized using a constant amplitude contour. An effect of this is to introduce occasional formant-frequency anomalies, including rates of change in the contour of inverted-frequency competitors that are more rapid than is typical for formant contours in natural speech.

There were seven conditions in the main experiment (see Table 1). In all cases, a scale factor of 50% was applied to the target formants. There was one control condition (C1), the stimuli for which contained the F2C (type = inverted, scale factor = 100%), but did not include the target F2. The opposite was true for the dichotic reference case (C7). The stimuli for the experimental conditions (C2–C6) contained all three target formants plus the F2C adjusted with a scale factor ranging from 0% to 100% in 25% steps. For each listener, the 42 sentences were divided equally across conditions (i.e., six per condition), such that there were always 19 keywords per condition. Allocation of sentences was counterbalanced by rotation across each set of seven listeners tested.[Table-anchor tbl1]

There were 45 sentences used in the training session (see Procedure in General Method). The training sentences consisted of three sets of 15, for which all the formants were scaled in parallel to 50%, 75%, or 100% depth; these sentences were intermingled in quasi-random order. The aim was to expose listeners to a range of depths of formant-frequency variation while maintaining reasonably high intelligibility. Immediately after the main session, there was a follow-up test in which listeners transcribed the set of 42 sentences (all scaled at 50% depth) once more, but this time under diotic presentation and without competitors.

#### Listeners and procedure

Twenty-one listeners (six males) completed the experiment successfully (mean age = 21.6 years, range = 18.4–34.8, *SD* = 4.4 years). As well as passing the training, listeners were required to meet a criterion of ≥ 50% keywords correct in the diotic follow-up task for their results to be included in the final dataset. In practice, there were no additional exclusions arising from the diotic follow-up task. None of the listeners took part in either of the other experiments. All stimuli in the training session and diotic follow-up were presented at a reference level of 72 dB SPL. In the main experiment, the reference level for the dichotic reference case (C7) was raised to 75 dB SPL, to compensate for the fall in loudness caused by splitting the formants between the two ears. Given that F1 is far more intense than the higher formants, the presentation level in the left ear when averaged across sentences was essentially at reference, whereas that in the right ear was ∼10 dB lower. For a given sentence, the level for each target formant (when present) was the same across conditions as for its counterpart in the dichotic reference case; the level of F2C was always matched to that of F2. There was some variation across sentences in the average level at each ear (±2–3 dB in the left), owing to differences in the intensity ratio between F1 and F2+F3, and also in the overall loudness of the stimuli, depending on the presence or absence of F2, F3, and the competitor formant.

### Results

**Figure 4 fig4:**
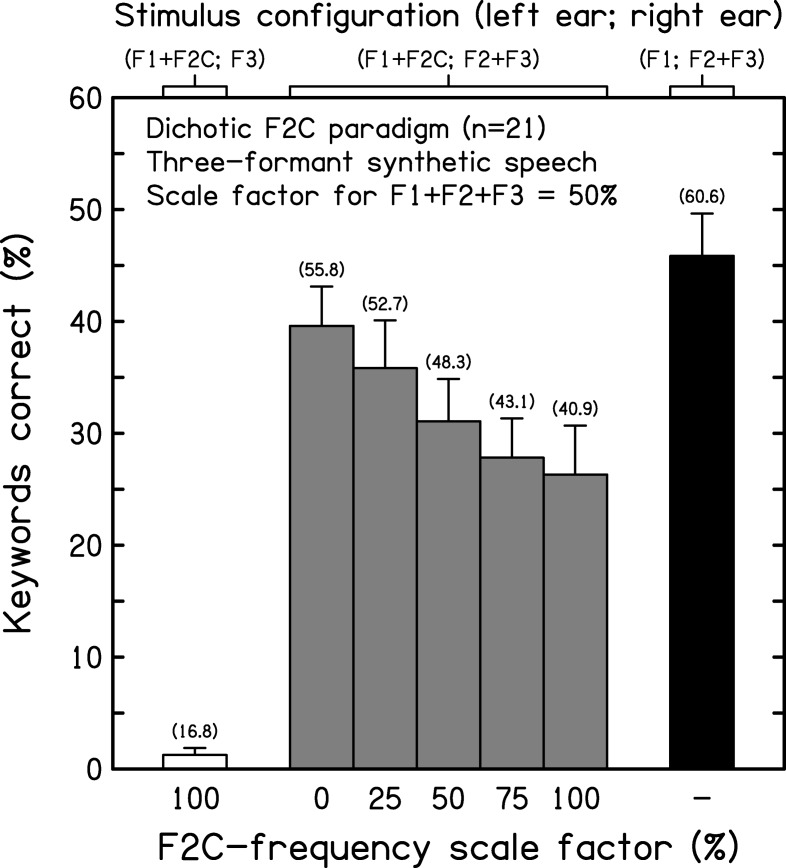
Results for Experiment 2: Influence of the depth of formant-frequency variation in competitor formants (F2Cs) on the intelligibility of synthetic-formant analogues of the target sentences. In this experiment, the frequency contour of F2C was always derived from that for F2 by inversion about the geometric mean (see main text). Mean keyword scores and intersubject standard errors (*n* = 21) are shown for the control condition (white bar), experimental conditions (gray bars), and the dichotic reference condition (black bar). The corresponding mean phonemic scores are shown in brackets above each bar. The top axis indicates which formants were presented to each ear; the bottom axis indicates the scale factor controlling the depth of formant-frequency variation in F2C (when present).

Figure 4 shows the mean keyword scores (and intersubject standard errors) across conditions. White, gray, and black bars indicate the results for the control, experimental, and dichotic reference conditions, respectively. The corresponding mean phonemic scores are shown in brackets above each bar. A one-way within-subjects ANOVA on the keyword scores showed a highly significant effect of condition on intelligibility, *F*(6, 120) = 26.946, *p* < .001, η^2^ = 0.574. The control condition (F1+F2C; F3) indicated that intelligibility was near floor when F2C was added full scale in the absence of the target F2. Pairwise comparisons indicated that the mean for the control condition differed from those for all other conditions (*p* < .001, in all cases). Clearly, F2C was not a good surrogate for the target F2 in supporting intelligibility. As expected, performance was best for the dichotic reference case.[Fig-anchor fig4]

An ANOVA restricted to the five experimental conditions (C2–C6) showed that the influence of scale factor on the effect of F2C on intelligibility was itself significant, *F*(4, 80) = 3.726, *p* = .008, η^2^ = 0.157. Adding a competitor with an inverted frequency contour to a target sentence typically reduced its intelligibility relative to the dichotic reference case. This reduction was least for 0%-depth (constant), intermediate for 50%-depth, and greatest for 100%-depth F2Cs (6.3, 14.8, and 19.6 percentage points, respectively). Once the scale factor applied to F2C was ≥ 25%, the reduction in keyword scores relative to the dichotic reference case was significant (range: *p* = .006 – < .001). Pairwise comparisons among the five experimental conditions indicated that the mean scores for the 0%- and 25%-depth cases were significantly different from those for the 75%- and 100%-depth cases (range: *p* = .040 – .002).

The decline in intelligibility as the scale factor for the inverted F2C increased was smooth and progressive; there was no sign of a minimum for the 50%-depth case. This pattern suggests that competitor efficacy depends on the overall depth of frequency variation in the contour of F2C, not its depth relative to that for the other formants (all set to 50% depth). Following Roberts et al. (2010), competitor efficacy may be defined as the impact on intelligibility of adding F2C in relation to performance for the dichotic reference condition (corresponding to 0% efficacy) and for the F1+F2C+F3 control condition (corresponding to 100% efficacy). By this definition, the mean efficacy of F2C varied from 14.0% (0%-depth case) to 43.8% (100%-depth case). The latter is similar to that estimated for the comparable sentence-length materials used by Summers et al. (2010) in their second experiment.

The mean diotic follow-up score for the 42 sentences used in the main experiment was 63.7%; this is very similar to that observed for comparable sentences in an earlier study (Summers et al., 2010), despite our use here of formant-frequency contours scaled to 50% depth. Diotic performance was better than the mean dichotic reference score of 45.9%, albeit with the caveat that listeners had already been exposed to degraded versions of these sentences during the main session. The intelligibility cost of dichotic presentation (17.8 percentage points) is in line with that previously reported for synthetic-formant analogues of similar sentence-length materials (10–20 percentage points; Summers et al., 2010). The corresponding mean phonemic scores for diotic and dichotic performance without competitors were 75.1% and 60.6%, respectively.

### Discussion

Sentence intelligibility is typically reduced when the target speech is accompanied by a competitor formant (F2C) generated using versions of the inverted frequency contour of F2 that were scaled to produce different mean depths of formant-frequency variation. The impact of the competitor on keyword recognition is unlikely to arise from energetic masking, because the F1 of the target sentence was lower in frequency and more intense than the competitor presented in the same ear. Therefore, the competitor’s effect must arise primarily through informational masking. The results confirm the finding that competitor efficacy is critically dependent on the time-varying properties of its frequency contour not only in the context of sine-wave speech (Roberts et al., 2010), but also in more speech-like simulations (Summers et al., 2012). Furthermore, competitors with inverted frequency contours are effective regardless of whether the inversion is around the geometric mean frequency of F2 or its spectral centroid (cf. Roberts et al., 2010). This merits note because the geometric mean of F2 is ∼100 Hz on average above its centroid, and the different point of inversion changes considerably the detail of a competitor’s frequency contour.

The key finding is that the competitor becomes progressively more effective as the depth of formant-frequency variation in F2C increases. This outcome is similar to that found for the effect of differences in rate of formant-frequency change in the competitor (Summers et al., 2012), where competitor efficacy increased for rates up to at least twice the baseline rate for the natural utterances used. The maximum depth of F2 variation in the competitors tested here was limited by the need to avoid close approaches or crossovers between adjacent formants in the ensemble, and as a consequence could not be set to exceed the unadjusted depth of F2 in the natural utterances. In the absence of competitors with greater F2 depth, the possibility that the 100%-depth competitors are the most effective because they match the natural depth of F2 cannot be ruled out. However, given the similarities between the present results and those of Summers et al. (2012), and given that manipulations of either the rate or depth of formant-frequency variation affect the velocity of this variation in the competitor, we contend that it is likely to be the overall extent of frequency variation in the competitor that governs its efficacy, rather than its similarity to the original depth.

If differences in the average modulation depth of formant-frequency contours are used as a cue to group and/or select formants, one might have expected to see evidence of tuning of the modulation-depth function around the 50% value to which all the target formants were scaled. Even if an effect of similarity were secondary to that of magnitude, one might still predict a local minimum in intelligibility around the 50% value. No such pattern was observed, albeit with the caveat that in principle tuning might be affected by the extent of frequency variation in F2C relative to that for all the target formants, not just F2. This factor might act to blur any effect of tuning, because different target formants vary in their original depth of frequency variation and so the extent of this variation may not always be most similar for F2C and F1 (or F3) when F2C is scaled by the same percentage as F1 and F3. Although not conclusive, the apparent absence of tuning in the effects of rate (Summers et al., 2012) and depth of formant-frequency variation is also consistent with the idea that the impact of an extraneous formant does not depend on speech-specific acoustical constraints. This interpretation is evaluated more directly in Experiment 3 through manipulation of the acoustic properties of F2C in ways that change its articulatory plausibility.

Notwithstanding the general points made in the beginning of this article about the ecological validity of our approach, another difference between our synthetic analogues and natural speech that merits comment is that all phonetic segments in our stimuli are represented as voiced (only buzz-excited formants were used) and on the same (constant) F0. In principle, these stimulus properties may encourage across-formant integration compared with more realistic approximations of speech involving both periodic and aperiodic excitation. Note, however, that these properties (unlike the dichotic configuration used) should not change the relative likelihood of integrating F2 or F2C into the phonetic percept.

## Experiment 3

Remez et al. (1994) and Remez (1996, 2001) interpreted their findings that an F2C created by time-reversing a tonal F2 was an effective competitor for sine-wave speech, but that a constant pure tone was not, in terms of the plausibility of speech-like variation. We explored the importance of speech-like variation for across-formant integration using an F2C whose frequency contour was simple, regular, and arbitrary. A contour derived from a periodic triangle wave was used, which we contend is not a speech-like pattern. The literature on precisely what constitutes speech-like variation in formant-frequency contours (or any other acoustic property) is very limited. As described below, our triangle-wave competitors were designed to have (at full scale) modulation rates and extents of frequency change similar to those found for formants in natural speech. This is important, given our findings thus far on the effects of rate and depth of formant-frequency variation.

A triangle wave differs from natural formant contours in having precise periodicity and a regular shape with consistently sharp peaks and troughs. Sharp peaks and troughs should be rare in the output of a dynamic system like the vocal tract, composed of articulators having mass and, when in motion, momentum. For example, Xu (2007) reported that the formant contours in natural utterances of repeated approximant-vowel sequences, such as “wawa . . .” and “yaya . . .,” show relatively rounded peaks and troughs that reflect the deceleration and change in direction of motion of the tongue, jaw, and lips. Despite the speech-likeness of inverted-frequency F2C contours being somewhat compromised by occasional relatively rapid frequency changes arising from errors in formant-frequency estimates (see above), the perceptual difference between inverted-frequency and triangle-wave F2Cs is compelling—the former sound like a potential component of a speech sound, but the latter do not.

### Method

#### Stimuli and conditions

**Figure 5 fig5:**
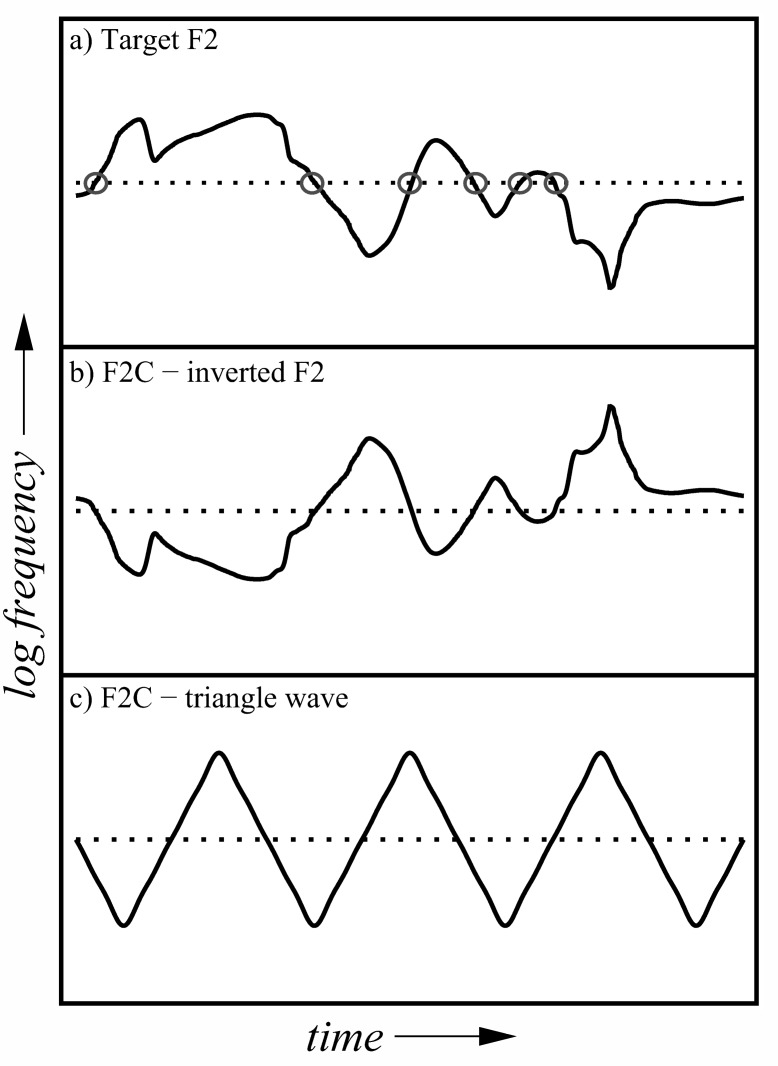
Stimuli for Experiment 3: Schematic illustrating how the formant-frequency contour of the triangle-wave F2C was derived from that of the target F2. Note the use of a log frequency scale in this figure. Using the example sentence “The mud was brown,” the top panel depicts the formant-frequency contour of the target F2 (solid line), its geometric mean frequency (dotted line), and zero crossings relative to the geometric mean (circles). The middle panel depicts the F2C whose frequency contour was derived from that of F2 by inversion about the geometric mean (a plausibly speech-like variation); the bottom panel depicts the frequency contour for the corresponding triangle-wave F2C (not plausibly speech-like). The triangle-wave frequency contour was generated using the first four odd harmonics of the chosen period for the triangle-wave function; the number of half cycles corresponds to the number of zero crossings plus one. For illustrative purposes, the starting phase in this example was not set randomly but was instead chosen to produce a negative-going contour whose starting (and ending) frequency corresponded to the geometric mean frequency of the target F2 contour (dotted line). The full set of scale factors used to control the depth of formant-frequency variation in the F2C ranged from 100% to 0%, in steps of 25%. The amplitude contours (not shown here) for the target formants and F2Cs are the same as described for Experiment 2.

This experiment used the same dichotic configuration as for Experiment 2 and was similar in overall design. The stimuli comprised synthetic analogues of 48 sentences; there was no overlap with the sentences used in the other experiments. A set of F2 competitors was created for each sentence in the main experiment. Figure 5 illustrates schematically the two types of frequency contour used for F2C in this experiment, and their relationship to that for the target F2. In one condition, the frequency contour of each F2C was created by inverting the frequency contour of the target F2 without rescaling (middle panel). For the other experimental conditions, F2C had the frequency contour of a triangle wave (bottom panel); the parameters for the triangle-wave frequency contour were chosen broadly to match the average rate and depth of variation for the target F2, and hence for its inverted F2C counterpart. In outline, the period was set in relation to zero crossings at the geometric mean frequency (see top panel) and the peak-to-trough range was matched to that of the target F2 on a log scale, but was made symmetrical by centering the range on the geometric mean. The triangle-wave contour was then rescaled to the desired depth. In greater detail, the process of generating the triangle-wave contours was as follows:[Fig-anchor fig5]

The triangle-wave frequency contour for F2C was generated using the first four odd harmonics of the general linear form of the triangle-wave function (see, e.g., Hartmann, 1998). This set of harmonics provides a contour with a characteristic triangular shape but the peaks and troughs are slightly smoothed, thus avoiding the synthesis artifacts which can be associated with rapid changes in the center frequency of a second-order resonator (see Summers et al., 2012). The amplitude of the triangle wave at time *t*, *a(t)*, is given by:

$$a(t)=\frac{8}{\pi^{2}}\sum_{n=1,3,5,7}\frac{1}{n^{2}}\cos(2\pi \;nt/T)$$ 2

where *n* is harmonic number, and *T* is set equal to the estimated modulation period (“cycle rate”) for the frequency contour of the target F2. This estimate was obtained by: (a) down-sampling the extracted F2 contour by averaging over 40 samples, which corresponds to low-pass filtering at 25 Hz; (b) computing the number of times plus one that the smoothed F2 contour crossed the geometric mean frequency, and taking this value as an estimate of the number of half-cycles of modulation completed for the whole F2 track; (c) dividing this value by two to obtain the desired number of cycles for the triangle wave (maintaining precision in half-cycles); and (d) dividing the duration of the F2 contour by the desired number of cycles. Performing these operations using the low-pass F2 contour reduced the likelihood of introducing spurious extra half cycles as a result of minor fluctuations in the F2 frequency contour close to its geometric mean. The triangle-wave function was then converted to a form suitable for generating the desired frequency contour for the triangle-wave F2C. The desired contour was triangular on a log frequency scale, with a peak-to-trough range equal to the maximum-to-minimum range for the whole target F2, but centered on a frequency equal to the geometric mean of the target F2 contour. This conversion, and the rescaling of the depth of frequency variation, was achieved using Equation 3. The center frequency for a triangle-wave F2C at time *t*, *f*(*t*), is given by:

$$f(t)=g+\exp\left(\frac{xra(t)}{\text{MAX}(a(t))}\right)$$ 3

where *x* (0 ≤ *x* ≤ 1) is the scale factor, *a*(*t*) is the triangle-wave function (Equation 2), *g* is the geometric mean of the frequency contour of the target F2, *r* is half the log-range of the target F2, and MAX(*a*(*t*)) is the maximum amplitude of the triangle-wave function. Note that division by MAX(*a*(*t*)) is needed to ensure that *a*(*t*) is scaled between −1 and 1, because the triangle-wave function is defined by a finite number of odd harmonics.

Once again, stimuli were selected such that the nominal frequency of F2C was always ≥ 80 Hz from that of F1 at any moment in time. The starting phase of the triangle wave was chosen randomly for each competitor and for each rotation of the experiment; also a new random choice was made on those occasions when the initial choice would have violated the constraint on the proximity of F2C and F1. Note that the geometric mean frequency of the full triangle-wave contour for F2C exactly matches that of the target F2 only when the complete track contains an even number of half cycles. Inevitably, there will be some deviation when there is an odd number of half cycles, the magnitude of which is dependent on starting phase and also declines as the number of complete cycles increases. However, this discrepancy was small in relation to the peak-to-trough range (RMS deviation = 11.2 Hz for the set of F2Cs used; *SD* = 1.2 Hz). Furthermore, there is evidence for a good deal of tolerance in the perceptual estimation of mean F2 frequency (e.g., Flanagan, 1955; Mermelstein, 1978), which suggests that competitor efficacy is likely to depend far less on the mean frequency of F2C than on the range of frequency variation. Indeed, the results of Mermelstein (1978) indicate a mean difference limen of about 185 Hz for the perceptual estimation of F2 in a dynamic context.

**Figure 6 fig6:**
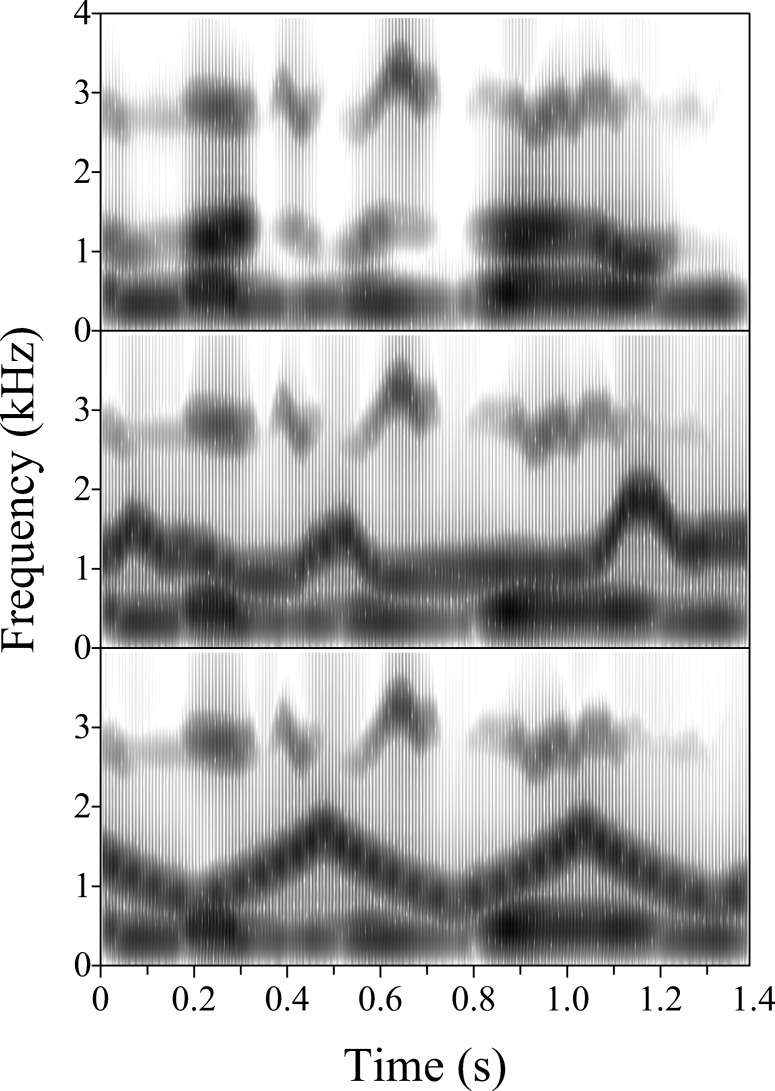
Stimuli for Experiment 3: Wideband spectrogram of a synthetic-formant analogue (F0 = 140 Hz) of the example sentence “The mud was brown” (top panel) and of two variants created by replacing the target F2 with a constant-amplitude competitor (F2C). The frequency contour of F2C was derived from that of F2 either by inversion about the geometric mean (middle panel) or by using a triangle-wave contour (bottom panel) whose rate and depth were matched to those of the target F2. The inverted F2C preserves a plausibly speech-like pattern of frequency variation, but the triangle-wave F2C does not. Note that the apparent distortion of the triangle-wave contour simply reflects the use here of a linear frequency scale. These spectrograms were created using stimuli for which the depth of formant-frequency variation was scaled to 50% for the target formants (baseline) and to 100% for F2C (maximum). For convenience, the ear of presentation used for the different formants is not represented here.

The amplitude contour of all F2Cs (whether inverted or triangle wave) was set to a constant value corresponding to the RMS power of the amplitude contour for the target F2. This is consistent with the method for Experiment 2 and also has the advantage of emphasizing the regular and arbitrary nature of the triangle-wave competitor. One outcome of using fixed-level continuous excitation is that there is, in effect, an onset asynchrony between F2C and the other formants when the first phonetic segment of the target sentence is of low amplitude. Note, however, that the perceptual contribution of one formant to a CV syllable is not reduced significantly unless there is an onset asynchrony of ∼300 ms relative to the other formants (Darwin, 1981), whereas the (effective relative) lead time on F2C for our stimuli rarely exceeded 100 ms. Longer offset asynchronies occurred for some of our stimuli, but their perceptual consequences are likely to have been modest compared with those of onset-time differences of similar magnitude (cf. Darwin, 1984; Darwin & Sutherland, 1984; Roberts & Moore, 1991). Figure 6 illustrates both types of F2C in the context of the target F1 and F3, using the wideband spectrogram of a synthetic analogue of an example sentence (top panel) and of two variants derived from it by replacing the target F2 with its inverted (middle panel) or triangle-wave counterpart (bottom panel). For convenience, the dichotic configuration used in the experiment to present the target formants and F2C is not represented here. Rather, the figure is intended to give a sense of the speech-likeness of frequency variation in the inverted and triangle-wave F2Cs, relative to that of the target F2.[Fig-anchor fig6]

There were eight conditions in the main experiment (see Table 2). As before, a scale factor of 50% was applied to all the target formants. The control condition (C1) was different from its counterpart in Experiment 2; here, the stimuli comprised F2 and F3 only. The new control was intended to provide a benchmark measure of intelligibility when F1 does not contribute perceptually to the sentence. The stimuli for the experimental conditions (C2–C7) contained all three target formants plus the F2C. For C2–C6, the frequency contour for F2C was a triangle wave adjusted with a scale factor ranging from 0% to 100% in 25% steps. For C7, the frequency contour for F2C was inverted and scaled to 100% depth; this is a direct counterpart of C6 in Experiment 2. The final condition was the dichotic reference case (C8). For each listener, the 48 sentences were divided equally across conditions (i.e., six per condition), such that there were always 18 or 19 keywords per condition. Allocation of sentences was counterbalanced by rotation across each set of eight listeners tested. The training session was the same as for Experiment 2, with the aim of exposing listeners to some reasonably intelligible stimuli with a range of depths of formant-frequency variation. A diotic follow-up test of intelligibility was not included in Experiment 3.[Table-anchor tbl2]

#### Listeners and procedure

Twenty-four listeners (five males) successfully completed the experiment (mean age = 21.2 years, range = 18.3–33.3, *SD* = 3.8 years). Eleven of the listeners also took part in Experiment 1. As well as passing the training, listeners were required (in the absence of a diotic follow-up) to meet an additional criterion for their results to be included in the final dataset—a mean score of ≥ 20% keywords correct in the main session when collapsed across all conditions. This nominally low criterion was chosen to take into account the poor intelligibility expected for some of the stimulus materials used in the main session. Four listeners were replaced based on this criterion. As for Experiment 2, stimuli in the training and main sessions were based on reference levels of 72 and 75 dB SPL, respectively.

### Results

**Figure 7 fig7:**
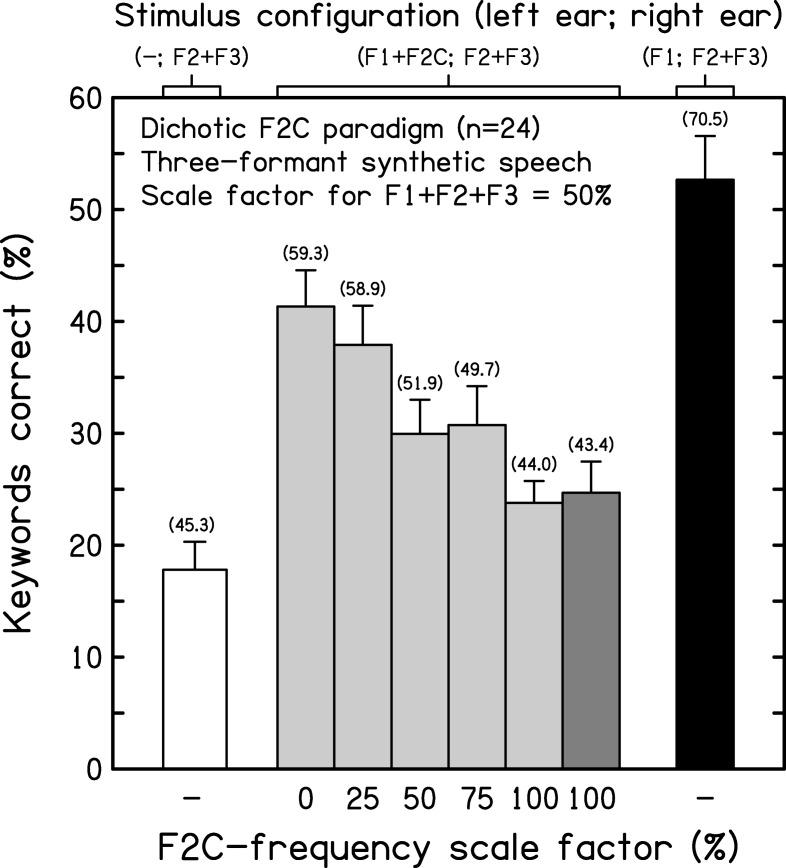
Results for Experiment 3: Influence of the depth of formant-frequency variation in competitor formants (F2Cs) on the intelligibility of synthetic-formant analogues of the target sentences. In most conditions, the frequency contour of F2C was a triangle wave whose rate and depth of variation were set in relation to those of the corresponding unscaled F2 (see main text), but in one condition the frequency contour of F2C was derived from that for F2 by inversion about the geometric mean. Mean keyword scores and intersubject standard errors (*n* = 24) are shown for the control condition (white bar), experimental conditions (triangle-wave cases = light gray bars; inverted case = dark gray bar), and the dichotic reference condition (black bar). The corresponding mean phonemic scores are shown in brackets above each bar. The top axis indicates which formants were presented to each ear; the bottom axis indicates the scale factor controlling the depth of formant-frequency variation for F2C (when present). Note that the control condition used here is different from the one used in Experiment 2.

Figure 7 shows the mean keyword scores (and intersubject standard errors) across conditions. White, gray, and black bars indicate the results for the control, experimental, and dichotic reference conditions, respectively; light and dark gray indicate the results for the triangle-wave F2C cases and for the 100%-depth inverted F2C comparison case, respectively. The corresponding mean phonemic scores are shown in brackets above each bar. A one-way within-subjects ANOVA on the keyword scores showed a highly significant effect of condition on intelligibility, *F*(7, 161) = 17.008, *p* < .001, η^2^ = 0.425. Relative to the dichotic reference case, intelligibility more than halved when F2+F3 were presented alone (control condition). Nonetheless, despite the absence of the target F1, performance remained substantially above chance (∼0%), *t*(23) = 7.12, *p* < .001. Pairwise comparisons indicated that the mean for the control case differed from those for all but one of the other conditions (range: *p* = .035 – < .001); the exception was the 100%-depth inverted F2C case (*p* = .06).[Fig-anchor fig7]

An ANOVA restricted to the six experimental conditions (C2–C7) showed that the influence of scale factor on the effect of F2C on intelligibility was itself significant, *F*(5, 115) = 7.893, *p* < .001, η^2^ = 0.255. Adding a competitor with a triangle-wave frequency contour to a target sentence typically reduced its intelligibility relative to the dichotic reference case. This reduction was least for 0%-depth (constant), intermediate for 50%-depth, and greatest for 100%-depth F2Cs (11.3, 22.7, and 28.9 percentage points, respectively). The reduction in keyword scores was significant for all F2Cs tested (range: *p* = .039 – < .001). Pairwise comparisons were performed to evaluate the relative effects of the five experimental conditions that used triangle-wave F2Cs. These indicated that the mean scores for the 0%-depth (constant) case differed from those for the 50%, 75%, and 100%-depth cases (range: *p* = .019 – < .001), and that the mean scores for the 100%-depth (full scale) case differed from those for all other cases (range: *p* = .041 – < .001).

Although the decline in intelligibility as the scale factor for the triangle-wave F2C increased shows a modest departure from monotonicity for the keyword scores at 50%- and 75%-depth (mean difference in keyword scores = 0.8 percentage points; *p* = .819), the overall effect of increased scale factor on keyword and phonemic scores suggests that, as in Experiment 2, competitor efficacy depends on the overall depth of frequency variation in the contour of F2C. The reduction in recognition performance arising from adding a 100%-depth inverted F2C (28.0 percentage points) is almost identical to that observed for the 100%-depth triangle-wave F2C. Furthermore, the impact of adding either type of full-scale F2C is to reduce performance to a level not much above that for the F2+F3 control case; indeed, mean phonemic scores were within 2 percentage points of their control counterpart. The implications of these outcomes are considered below. It is also worth noting that the mean number of phonemes transcribed by listeners does not decline greatly even when correct identification of phonemes falls to relatively low levels, as for the F2+F3 control case.

In terms of our measure of competitor efficacy, the absence of an F1+F2C+F3 control condition means that changes in performance across the experimental conditions can only be used to compute minimum estimates of efficacy—that is, when a value of zero is assumed for the control case. These estimates varied from 21.5% to 54.9% (0%- and 100%-depth cases). In practice, these values would have been higher, as the score for the missing control case would have been nonzero. Nonetheless, both estimates are greater than the corresponding values from Experiment 2. Most probably, this is related to differences in baseline intelligibility for the nonoverlapping sets of target sentences used in the two experiments.

### Discussion

Contrary to the argument that across-formant grouping and/or selection depends on speech-specific acoustical constraints (e.g., Remez, 1996, 2001; Remez et al., 1994), the triangle-wave competitors were as effective as their more speech-like counterparts. It should be acknowledged that there is arguably more formant-frequency variation in a triangle-wave F2C than in its inverted counterpart, because the maximum peak-to-trough difference is almost always reached more than once. Nonetheless, it is clear that a substantial deterioration in performance can be produced by a pattern of formant-frequency variation that is not plausibly speech-like. Although not typically formulated to deal specifically with stimuli involving competition, popular theories of speech perception do not predict this result. If speech perception involves recovery of the phonetic gestures of the talker (see, e.g., Fowler, 1986; Liberman, 1982) and is thus able to exclude an extraneous formant perceptually on the basis of the implausibility of the underlying articulatory gestures, one would have expected the triangle-wave competitors to be less effective than the inverted-contour competitors. Furthermore, statistical pattern-matching accounts of speech perception operating on acoustical similarities in time-varying patterns without reference to articulation (e.g., Diehl, Lotto, & Holt, 2004) would also be likely to predict that speech-like competitors are more difficult to segregate from the target formants than nonspeech-like ones.

The finding in both experimental conditions using 100%-depth F2Cs (triangle wave and inverted) that performance was not much better than for the F2+F3 control is consistent with the suggestion that F1 may have been largely excluded from the percept of the target sentences. This idea was investigated by an examination of changes across conditions in the likelihood of making particular classes of phonemic response. Any effect of the competitor on perceptual evaluation of the F1 frequency might be expected to manifest as errors in judgments of vowel height (cf. Nearey & Levitt, 1974), but there was no evidence of systematic effects on vowel judgments in the pattern of phonemic responses. However, there are grounds for doubting whether this approach is useful for evaluating the F1 capture hypothesis, because even the physical absence of F1 (F2+F3 control) did not increase the proportion of high-F1 vowels reported (as would occur if F2 were interpreted as F1). Nonetheless, according to a grouping account, tuning by similarity in the depth of frequency variation—for which our data provide no evidence—would be predicted regardless of whether F2C acted as an alternative to F2 or through the perceptual capture of F1 (thus disrupting its integration with F2+F3 in the other ear).

## General Discussion

### Perceptual Consequences of Frequency Variation in an Extraneous Formant—Competitor Effects on Across-Formant Grouping and Informational Masking

Intelligibility typically falls when a target sentence is accompanied by a competitor with a time-varying frequency contour. Given that the dichotic configuration we used largely controls for energetic masking of the target formants by the competitor, this effect must arise primarily from informational masking. Our main motivation for using the F2C paradigm in our previous studies was to explore the factors influencing across-formant grouping, but the impact of an extraneous formant on sentence intelligibility might also arise from limitations on a range of other perceptual and cognitive processes. Here, we take a broader perspective to evaluate our current results and to reinterpret some aspects of the findings from two of our previous studies (Roberts et al., 2010; Summers et al., 2012).

#### Absence of evidence for a grouping primitive based on similarity in dynamic properties

A typical informational-masking experiment involves the presentation of one or more target tones embedded in a sequence of masking tones and/or a set of temporally overlapping masking tones. The properties of these “context” tones, (e.g., their frequency distribution) are generally selected to reduce or eliminate energetic masking of the target tone(s). Studies of this kind have shown that increases in masker (or target) uncertainty—for example, in the extent of frequency variation—can greatly increase thresholds for target detection, discrimination, or identification (for reviews, see Kidd et al., 2008; Watson, 2005). Although much of the research focus has concerned masker uncertainty, over the past 20 years or so several studies have shown that informational masking can be reduced by introducing grouping cues that assist the perceptual segregation of the target from the masker. For example, segregation cues such as onset asynchrony, spatial separation, and qualitative differences between the target and masker can lead to a substantial release from informational masking in detection and discrimination tasks (e.g., Durlach et al., 2003b; Lee & Richards, 2011; Neff, 1995). Even more germane to the current experiments, segregation benefits are also apparent in suprathreshold contexts where target identification is required, such as identifying distinctive arbitrary patterns (Kidd et al., 1998) or the calls of songbirds (Best et al., 2005) in the presence of interfering sounds.

There are many dimensions on which targets and maskers can differ perceptually, not all of which necessarily act as grouping constraints. The studies cited above all made use of relatively simple qualitative differences, such as a narrowband-noise target in a multitone masker (Neff, 1995) or a target tone with a frequency sweep in the opposite direction to that of the masking tones (Durlach et al., 2003b). What pattern of results might be regarded as the “signature” of a segregation cue based on target-masker dissimilarity in the context of the formant-competitor paradigm? One possibility would be a demonstration that the addition of further acoustic elements designed to capture the competitor can increase the intelligibility of the target sentences. However, the likelihood of observing this outcome experimentally is low, given that the additional elements are themselves likely to act as interferers. We contend that convincing evidence of the operation of a grouping constraint would also be provided by evidence of tuning—that is, informational masking should peak when the target sentence and the competitor are most similar on a particular dimension. This is precisely the pattern obtained when a well-established segregation cue, ΔF0, is used to distinguish the target and competitor formants (Summers et al., 2010). It is not, however, the pattern obtained when targets and competitors differ in the depth (current study) or rate (Summers et al., 2012) of formant-frequency variation; rather an increase in depth or rate for the competitor increases the impact on intelligibility.

The results obtained argue against the suggestion by Roberts et al. (2010) that the greater impact on intelligibility of competitors with time-varying frequency contours may reflect the operation of a grouping primitive based on similarity in the dynamic properties of broadband sounds. Furthermore, our failure to find evidence that the auditory system can use differences in the depth and rate of formant-frequency variation to segregate formants is consistent with current assertions that there are only a few genuine primitives governing concurrent sound segregation, such as temporal synchrony (e.g., Shamma et al., 2011; Shamma & Micheyl, 2010) and harmonicity (Micheyl et al., 2013). Instead, we propose that our results indicate a progressive rise in informational masking of the target sentence as the total frequency variation in the competitor increases, as will occur when either the depth or the rate of its formant-frequency variation is increased.

#### The relationship between frequency variation in a masker and informational masking

We are aware of only two studies concerned explicitly with the effects of rate of masker frequency variation on the informational masking caused by that masker. Both studies used speech stimuli and provided evidence that the impact of informational masking on the recognition of target speech was greatest for the fastest masker speech rate tested (Chen et al., 2008; Summers et al., 2012). Furthermore, to our knowledge there are no studies concerned explicitly with the effects of depth of masker frequency variation on informational masking, either for speech or nonspeech stimuli. What is clear is that, in the absence of an effective segregation cue (e.g., ΔF0), the F2C paradigm offers considerable scope for informational masking, owing to the considerable degree of unpredictability of the frequency contours of the target and competitor formants.

The properties of stimuli in the F2C paradigm differ in important ways from those typically used in studies of informational masking. In such research, it is usual to employ narrowband targets and broadband maskers, and for increases in the extent of frequency variation across time to be confounded with decreases in overall spectro-temporal coherence, arising from larger discontinuities between successive segments of the stimulus. For example, maskers (and targets) are often constructed by concatenating a sequence of multiple tone bursts such that frequency variation across time is generated by making an independent random draw of frequencies for each successive burst (e.g., Kidd et al., 1994). In contrast, our experiments involve changing the extent of frequency variation in the target and competitor formants while preserving formant-frequency contours with coherent trajectories. Also, successful sentence recognition involves adequate integration across frequency—and ear—of the phonetically relevant information carried by the three target formants.

Of possible relevance in this context is the phenomenon of frequency modulation detection interference (FMDI), in which the detection (or discrimination) of FM on one carrier frequency can be impaired by the presence of FM on another carrier frequency, even when the carriers are widely separated in frequency (Wilson et al., 1990). FMDI acts in both directions, unlike the upward spread of energetic masking, and so in principle the formant-frequency variation in F2C might affect processing of the formant-frequency variation in F1. Consistent with this notion, the threshold for detection of FM in the center frequency of a formant-like harmonic complex is elevated in the presence of a similar harmonic complex in a higher or lower frequency region when the center frequency of the masking complex is also frequency modulated; no FMDI is observed for constant-frequency maskers (Lyzenga & Carlyon, 1999). Similar, but smaller, effects are found for contralateral maskers (Lyzenga & Carlyon, 2000), and so in principle F2C might also affect extraction of target F2 properties. Comparable results are found for the detection and discrimination of formant-like frequency glides (Lyzenga & Carlyon, 2005); these stimuli approximate more closely the patterns of formant-frequency variation typically found in natural utterances.

FMDI studies are concerned only with changes in thresholds for modulation detection or discrimination. In the suprathreshold context of our stimuli, it is not obvious why a slight elevation in FMDI threshold for the target formants (arising from the presence of F2C) should reduce our ability to extract the phonetically relevant time-varying properties of the target formants. Nonetheless, there are clear parallels between the results of these FMDI studies and our findings, particularly the idea that increasing the extent of frequency variation in F2C might make less salient or available the phonetic information carried by the target formants, including those presented in the opposite ear. There is now a growing body of evidence that a wide range of factors that increase the cognitive load on listeners impair the sensory analysis of speech signals (see, e.g., Mattys et al., 2009, 2012; Mattys & Wiget, 2011). In the context of the current study, one way in which this may happen is through the capture of attentional resources by the extraneous formant, on the basis that the interferer becomes harder to ignore as the extent of its formant-frequency variation increases.

### Absence of Evidence for Speech-Specific Acoustical Constraints on Grouping

Research during the 1950s, 60s, and 70s—most notably at the Haskins Laboratories—revealed the nonlinear and noninvariant nature of the acoustical cues to phonemic identity in speech perception, and the complex ways in which these cues are shaped by constraints on the articulatory gestures that produce them (e.g., Cooper et al., 1952; Liberman et al., 1967). This body of work led some theorists to propose that speech perception cannot be explained in terms of the general processes of auditory perception, but rather that phonetic information is perceived by a system specialized to detect the intended articulatory gestures of the talker (e.g., Fowler, 1986; Liberman & Mattingly, 1985). It has also been argued that phonetic perception has precedence over nonspeech processes, in that it is not subject to the constraints of general-purpose auditory grouping cues when extracting phonetic elements from an acoustic signal (e.g., Whalen & Liberman, 1987; but see Bailey & Herrmann, 1993, for a critique of their study). By this account, the ability to extract a coherent set of phonetic elements from an acoustic signal depends on speech-specific grouping constraints—namely, the articulatory plausibility of the acoustic elements being extracted (e.g., Remez, 2003, 2005; Remez et al., 1994).

The importance of articulatory information, and its plausibility, for the perceptual organization of speech has often been asserted and assumed. However, what this means in terms of critical acoustical correlates has not been considered in any detail (see, e.g., Darwin, 2008), let alone subjected to rigorous testing. Our finding that the impact of a competitor formant on sentence intelligibility depends on the extent of its frequency variation, but not the articulatory plausibility of this variation—or its speech-likeness without reference to articulation—implies that neither across-formant grouping nor the attention-driven selection of formants from a stimulus ensemble are governed by speech-specific acoustical constraints. This outcome is consistent with a growing body of evidence on the speech processing abilities of a variety of nonhuman species, including chimpanzees (Heimbauer et al., 2011), rats (Ahmed et al., 2011), and budgerigars (Welch et al., 2009). These studies suggest that the perception of speech sounds does not necessarily involve specialized articulatory knowledge.

### Concluding Remarks

The results confirm and extend those of our previous research on the effects of extraneous formants on the intelligibility of target speech (Roberts et al., 2010; Summers et al., 2010, 2012). Adding competitor formants with time-varying frequency contours typically reduces intelligibility; in the context of the dichotic F2C paradigm, this effect is one of informational rather than energetic masking. The impact on intelligibility of depth of formant-frequency variation in the competitor is not tuned to target-competitor similarity on this dimension, but rather increases as the depth of variation increases. This pattern differs from the tuned response observed for a known primitive grouping cue (ΔF0; Darwin, 1981; Summers et al., 2010), but is similar to that found for the rate of formant-frequency variation (Summers et al., 2012). Taken together, these outcomes indicate that across-formant grouping is not governed by similarity in the dynamic properties of the formant-frequency contours. Rather, an extraneous formant more effectively corrupts or disrupts extraction of the phonetic properties of the target speech as the extent of frequency variation in that formant increases. Plausibly, this interference may have either a more specific cause—for example, a failure to exclude the acoustic variation in the extraneous formant from the perceptual evaluation of the target sentence—or a more general one—for example, a greater cognitive load on the listener as the extent of frequency variation in the competitor increases (cf. Mattys et al., 2012). Distinguishing these hypotheses using sentence-length utterances will be challenging, but it may be possible with shorter materials such as CV syllables (cf. Porter & Whittaker, 1980). Perhaps most notably, competitor efficacy appears not to depend on the plausibility of the articulatory movements implied by F2C. Hence, we can conclude that there are at least some circumstances—specifically, those involving informational masking by a single formant—in which the ability to attend selectively the formants of target speech and to ignore extraneous formants does not depend on speech-specific acoustical constraints. Of course, this outcome for acoustic-phonetic cues does not imply an absence of *linguistic* (e.g., lexical-semantic) constraints on speech perception under adverse listening conditions (see, e.g., Davis & Johnsrude, 2007; Mattys et al., 2012).

## Supplementary Material

10.1037/a0036629.supp

## Figures and Tables

**Table 1 tbl1:** Stimulus Properties for the Conditions Used in Experiment 2 (Main Session)

Condition	Stimulus configuration	Scale factor for F2C (relative to unscaled target F2)
	(left ear; right ear)	
C1	(F1+F2C; F3)	100%
C2	(F1+F2C; F2+F3)	0%
C3	(F1+F2C; F2+F3)	25%
C4	(F1+F2C; F2+F3)	50%
C5	(F1+F2C; F2+F3)	75%
C6	(F1+F2C; F2+F3)	100%
C7	(F1; F2+F3)	—
*Note.* The frequency contour of the competitor (F2C), when present, is inverted. The scale factor for F2C refers to the depth of variation in formant frequency, relative to that for the unscaled target F2. A scale factor of 0% indicates a constant frequency contour for F2C, corresponding to the geometric mean frequency of the target F2. The amplitude contour for F2C was set to a constant value. The target formants were scaled to 50% of their original depths in all conditions.		

**Table 2 tbl2:** Stimulus Properties for the Conditions Used in Experiment 3 (Main Session)

Condition	Stimulus configuration (left ear; right ear)	Type of frequency contour for F2C	Scale factor for F2C (relative to unscaled target F2)
C1	(—; F2+F3)	—	—
C2	(F1+F2C; F2+F3)	T	0%
C3	(F1+F2C; F2+F3)	T	25%
C4	(F1+F2C; F2+F3)	T	50%
C5	(F1+F2C; F2+F3)	T	75%
C6	(F1+F2C; F2+F3)	T	100%
C7	(F1+F2C; F2+F3)	I	100%
C8	(F1; F2+F3)	—	—
*Note.* The frequency contour of the competitor (F2C), when present, is either a triangle wave (T) or inverted (I). The scale factor refers to the depth of variation in formant frequency, relative to that for the unscaled target F2. A scale factor of zero indicates a constant frequency contour for F2C, corresponding to the geometric mean frequency of the target F2. The amplitude contour for F2C was set to a constant value. The target formants were scaled to 50% of their original depths in all conditions.			

## Footnotes

1 Strictly speaking, formants are acoustical resonances of the vocal tract whose effects are manifest as peaks in the spectral envelope of speech sounds. Nonetheless, these spectral prominences are themselves commonly referred to as formants.

2 The term “dynamic” is commonly used to refer to acoustic change over time, but it is rarely used accurately as referring specifically to motion produced by force. Rather, the term “kinematic” is preferable for our meaning, because it refers to motion without specific reference to force. However, the term “kinematic” is often used specifically in the context of describing articulator movement, and so to avoid confusion we use the term “dynamic” throughout.

3 ANOVAs were computed using SPSS (PASW Statistics 18, IBM Corp.). The measure of effect size reported here is partial eta squared (η^2^).
