# Supplementary material for: Formant-Frequency Variation and Informational Masking of Speech by Extraneous Formants: Evidence Against Dynamic and Speech-Specific Acoustical Constraints
Source: J Exp Psychol Hum Percept Perform. 2014 May 19;40(4):1507–25. doi: 10.1037/a0036629 (PMC4120706; doi:10.1037/a0036629)
Supplement: Supplementary file 1 [file XHP-XHP2-Roberts20131211-RR-F8.zip › Roberts_Summers_Bailey_Additional_Content.pdf]

Additional content for *"Formant-frequency variation and informational masking of speech by extraneous formants: Evidence against dynamic and speech-specific acoustical constraints,"* by Brian Roberts, Robert J. Summers, and Peter J. Bailey.

The audio files accompanying this document are examples of the stimuli whose construction is described fully in the main article.

Experiment 1:

Example sentence (keywords in bold): *"She was **reading** the **map**"*

Three-formant synthetic analogues. F0 frequency = 140 Hz. Diotic presentation with no competitor formant. 11 conditions.

Filename		Scaling of formant-frequency variation
bkbp_0458_000.wav	=	0% depth (constant)
bkbp_0458_010.wav	=	10% depth
bkbp_0458_020.wav	=	20% depth
bkbp_0458_030.wav	=	30% depth
bkbp_0458_040.wav	=	40% depth
bkbp_0458_050.wav	=	50% depth
bkbp_0458_060.wav	=	60% depth
bkbp_0458_070.wav	=	70% depth
bkbp_0458_080.wav	=	80% depth
bkbp_0458_090.wav	=	90% depth
bkbp_0458_100.wav	=	100% depth (natural)

Experiment 2:

Example sentence (keywords in bold): *"The **ladder lay on** the **floor**"*

Dichotic presentation with competitor (F2C). 7 conditions.

F1, F2, and F3 with formant-frequency variation scaled to 50% depth.
F2C = Inverted F2-frequency contour, constant amplitude.

Filename		(Left ear; Right ear)	
bkbp_1295_C1.wav	=	(F1+F2C; F3)	; F2C at 100% depth
bkbp_1295_C2.wav	=	(F1+F2C; F2+F3)	; F2C at 0% depth
bkbp_1295_C3.wav	=	(F1+F2C; F2+F3)	; F2C at 25% depth
bkbp_1295_C4.wav	=	(F1+F2C; F2+F3)	; F2C at 50% depth
bkbp_1295_C5.wav	=	(F1+F2C; F2+F3)	; F2C at 75% depth
bkbp_1295_C6.wav	=	(F1+F2C; F2+F3)	; F2C at 100% depth
bkbp_1295_C7.wav	=	(F1; F2+F3)	

Experiment 3:

Example sentence (keywords in bold): *"The **mud** was **brown**"*

Dichotic presentation with competitor (F2C). 8 conditions.

F1, F2, and F3 with formant-frequency variation scaled to 50% depth.

C2–C6: F2C = Triangle-wave frequency contour, constant amplitude.
C7: F2C = Inverted F2-frequency contour, constant amplitude.

<u>Filename</u>		<u>(Left ear; Right ear)</u>	
bkbp_1515_C1.wav	=	(–; F2+F3)	
bkbp_1515_C2.wav	=	(F1+F2C; F2+F3)	; F2C at 0% depth
bkbp_1515_C3.wav	=	(F1+F2C; F2+F3)	; F2C at 25% depth
bkbp_1515_C4.wav	=	(F1+F2C; F2+F3)	; F2C at 50% depth
bkbp_1515_C5.wav	=	(F1+F2C; F2+F3)	; F2C at 75% depth
bkbp_1515_C6.wav	=	(F1+F2C; F2+F3)	; F2C at 100% depth
bkbp_1515_C7.wav	=	(F1+F2C; F2+F3)	; F2C at 100% depth
bkbp_1515_C8.wav	=	(F1; F2+F3)	

This document was created on 10th March 2014
